# Sex Steroid-Mediated Control of Oviductal Function in Cattle

**DOI:** 10.3390/biology7010015

**Published:** 2018-02-02

**Authors:** Mario Binelli, Angela Maria Gonella-Diaza, Fernando Silveira Mesquita, Claudia Maria Bertan Membrive

**Affiliations:** 1 Department of Animal Sciences, University of Florida, PO Box 110910, Gainesville, FL 32611, USA; 2 Departamento de Reprodução Animal, Faculdade de Medicina Veterinária e Zootecnia, Universidade de São Paulo, Rua Duque de Caxias Norte, 255, Bairro: Jardim Elite, Pirassununga 13635-900, SP, Brazil; angela.gonella@usp.br; 3 Curso de Medicina Veterinária, Universidade Federal do Pampa, UNIPAMPA, BR 472-Km 592, Uruguaiana 97508-000, RS, Brazil; fernandomesquita@unipampa.edu.br; 4 Faculdade de Ciências Agrárias Tecnológicas—FCAT, Universidade Estadual Paulista “Júlio de Mesquita”, Rodovia Comandante João Ribeiro de Barros (SP 294), Km 651, Dracena 17900-000, SP, Brazil; cbertan@dracena.unesp.br

**Keywords:** estradiol, progesterone, fertility, gene expression, morphology

## Abstract

In cattle, the oviduct is a tubular organ that connects the ovary and the uterus. The oviduct lumen stages a dynamic set of cellular and molecular interactions to fulfill the noble role of generating a new individual. Specific anatomical niches along the oviduct lumen provide the appropriate microenvironment for final sperm capacitation, oocyte capture and fertilization, and early embryo development and transport. To accomplish such complex tasks, the oviduct undergoes spatially and temporally-regulated morphological, biochemical, and physiological changes that are associated with endocrine events of the estrous cycle. Specifically, elevated periovulatory concentrations of estradiol (E2) and progesterone (P4) influence gene expression and morphological changes that have been associated positively to fertility in beef cattle. In this review, we explore how E2 and P4 influence oviductal function in the beginning of the estrous cycle, and prepare the oviductal lumen for interactions with gametes and embryos.

## 1. Introduction

When Gabriele Falloppio first described and named the “small trumpets” as fallopian tubes in 1561, it is unlikely that he would have anticipated the relevance of such delicate structures for the reproductive biology field. Fallopian tubes in women, or oviducts in domestic animals, were considered for many years as only a “pipeline” for the transit of oocytes [1]. However, it is now well accepted that the oviduct plays a major role in sperm storage and capacitation [2,3,4], fertilization [5,6], and early embryo development [7,8]. However, gamete final maturation, fertilization, and early embryo development can be conducted artificially under laboratory conditions. The success of commercial in vitro embryo production contributed to the idea that the oviduct is merely a conduit for the transit of gametes and embryos [9]. However, multiple studies suggest that the oviduct participates actively in the reproductive process, engaging in biochemical communications with gametes and embryo as they migrate in its lumen. For example, the oviduct distinguishes between unfertilized oocytes and viable embryos in mares, retaining oocytes at the utero-tubal junction (UTJ), while driving embryos towards the uterine lumen [10]. Similar observations by Wetscher et al., 2005 [11] demonstrated that bovine embryos of different qualities and developmental stages show distinct migration patterns after intra-tubal embryo transfer. Additionally, oviductal tissue has been demonstrated to respond differently at the transcriptional level when exposed to X- or Y chromosome-bearing spermatozoa in gilts [12], as well as to single embryos in beef cattle [13].

Currently, it is well accepted that the oviductal environment plays a major role on embryonic developmental capacity. In particular, improved embryo development after culture with oviduct epithelial cells supports a relevant role of the oviductal epithelium up to around the fourth day after fertilization [14,15,16,17,18]. This corresponds to the period during which embryos are in transit through the oviduct. Strong evidence exists to indicate a greater quality and developmental potential of in vivo-produced embryos in comparison to their in vitro counterparts [19,20,21,22]. Such a difference is also expressed at the molecular level as indicated by distinctly different embryonic gene expression [19,23,24] and epigenome patterns [6,25] between in vivo and in vitro-produced embryos. In this regard, Rizos et al. [19] compared in vivo and in vitro produced embryos, and reported an aberrant transcription pattern of genes known to be involved in apoptosis, oxidative stress, gap junctions, and differentiation. These are molecular functions critical for embryo production. Salilew-Wondim et al. [25] compared the DNA methylation patterns and transcriptome of bovine embryos cultured in vitro, and then transferred to recipients at the zygote, 4-cell, or 16-cell stages, along with blastocysts produced entirely in vitro. It was observed that the longer the embryo is exposed to the in vitro culture, the greater the number of both hypermethylated and hypomethylated genomic regions. These epigenomic modifications were evenly distributed throughout the genome, and were detected in gene bodies and promoter regions. In addition, gene ontology analysis indicated that differentially methylated regions were found to affect several biological functions (i.e., ATP binding, apoptosis, glycolysis, genetic imprinting, chromosome segregation). Thus, those major molecular and functional contrasts between embryos exposed to in vitro versus in vivo conditions explain the differences in embryo quality. Most likely, in vitro conditions cannot yet properly recapitulate the in vivo environment [6]. This implicates the oviduct as a major regulator of embryo quality.

In vivo, during the window encompassed by endocrine events preceding the oocyte–oviduct interaction, as well as those taking place up to the oviduct–uterus transition by the embryo, the oviductal tissue is exposed locally and systemically to drastic changes of the ovarian steroid hormonal profile [26,27]. Scarce information is available regarding the regulation of oviductal function by estradiol (E2) and progesterone (P4), and it is mostly related to the mechanism of sperm/oocyte/embryo transport and interaction with oviductal cells (for reviews, please see [26,28,29,30,31,32,33,34]), and formation of the oviductal fluid (for reviews, please see: [9,35,36,37,38,39,40]). Nonetheless, several studies have shown that the elevated periovulatory concentrations of both hormones are positively associated to fertility in beef cattle [41,42,43,44]. In the present review, we explore how ovarian sex steroids, E2 and P4, influence oviductal functioning at the beginning of the estrous cycle, and prepare the oviductal lumen for the passage of gametes and embryos.

**Figure 1:**
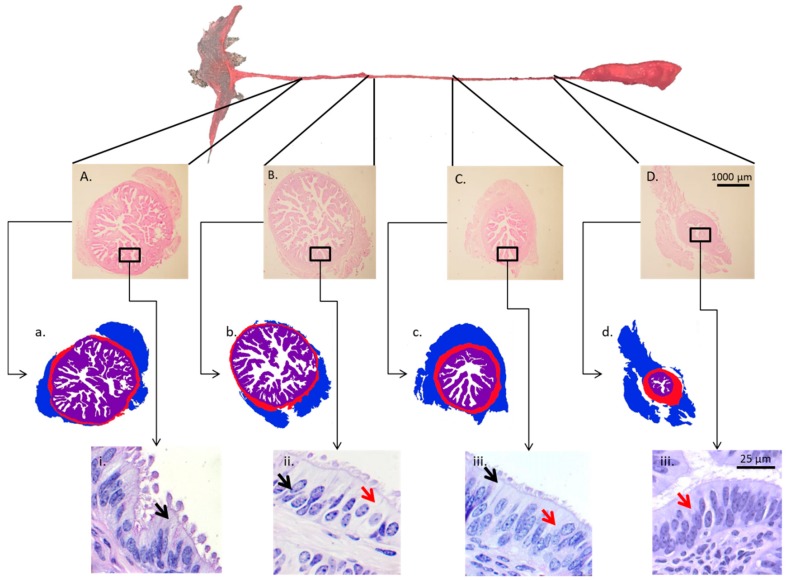
In the top, the gross anatomy of the cow oviduct is shown. Then, transversal sections of the deferent regions of the oviductal wall are shown (hematoxylin–eosin-stained tissues sections). Structural layers were digitally-colored to show the difference in the thickness of the tunicae between the regions (blue: tunica serosa; red: tunica muscularis; purple: tunica mucosa). At the bottom, microphotographs of the oviductal luminal epithelium, note the difference in cellular populations among regions (periodic acid–Shiff-stained tissue sections). A, a, and i: cranial segment of the ampulla; B, b, and ii: caudal segment of the ampulla; C, c, iii: cranial segment of the isthmus; D, d, iv: caudal segment of the isthmus; Black arrows: secretory cells; Red arrows: ciliated cells. Pictures are from the oviduct of a single animal that was in metestrus (recent ovulation).

To properly discuss sex-steroid control of oviductal function, particularities of oviductal biology must be put forth. First, the oviduct has different regions that fulfill specific functions [45]. Based on its macro-anatomical characteristics, the oviduct can be divided into four regions: the infundibulum, the ampulla, the isthmus, and the UTJ (Figure 1). The infundibulum contains fimbriae that pick up the oocyte soon after fertilization and lead it to the lumen of the ampulla. In the remarkably secretory ampulla, the oocyte completes nuclear and cytoplasmic maturation, followed by fertilization and the first cell divisions of the embryo. The UTJ and isthmus play major roles prior to fertilization, initially by retaining and capacitating spermatozoa [4]. After fertilization, they interact with the developing embryo as it is transported into the uterus, at the stage of 8–16 cells in cattle [26,46]. Second, in monovulatory species, such as cattle, the sex steroid concentrations to which oviductal cells are exposed vary according to the spatial relationship with the ovulating ovary (i.e., ipsilateral vs. contralateral) [47,48]. Third, the oviduct is an organ of difficult access. Unlike the ovary and uterus that can be more easily probed, collection of oviductal samples generally requires surgery or slaughter of animals. This generates a major restriction in the number of studies and number of animals/study, and results in a slow progress in the field of oviduct biology [9,33]. Finally, in vitro culture systems of epithelial oviduct cells have not yet been properly established. Whether cultured as monolayer, in co-culture, or suspension, studies have not been able to maintain oviduct cell morphology, and gene and protein expression comparable to in vivo conditions. Numerous research groups have dedicated major efforts towards the establishment of appropriate oviductal cell culture conditions; however, no consensus regarding a minimally ideal system, that properly reflects the in vivo situation, has been reached [49,50,51]. This represents a major obstacle to deepening the understanding of cellular and molecular mechanisms taking place in the organ.

In this context, the present review will explore the biology of sex steroids and their receptors, the influence of sex steroids in the abundance of oviductal transcripts and proteins, and the association between sex steroids and morphological characteristics of the oviduct that are likely to modulate fertility. Due to limitations in space, the present review focused on the oviductal luminal compartment. The muscular compartment plays critical roles in oviductal functions regulated by sex steroids and they deserve to be discussed in a future review.

## 2. Ovarian Sex Steroids and Their Receptors

A major advance in the field of reproductive endocrinology, initially based upon anatomical and histological evidence, was the proven theory that menstruation was under the regulation of an ovary-derived hormone [52]. The work by Edgar Allen and Edward Doisy published in 1923 [53] challenged the current hypothesis at the time, which had the corpus luteum (CL) as the source of the ovarian endocrine factor. By establishing a new method for testing the impact of ovarian extracts on reproductive tissues, and showing that follicular extract administration induced sexual maturity in rats, the authors paved the road for the discovery of E2 and P4 [53]. As a consequence, in 1929, Alfred Butenandt and Edward Doisy independently purified and crystallized estrone [54,55], a work that awarded Dr. Butenandt the Nobel Prize. P4 is a cholesterol-derived steroid hormone that can be synthesized and secreted mainly by corpora lutea in the ovaries, but also in the testis, placenta, and the adrenal glands, or de novo synthesized in several tissues of the nervous system [55]. E2 has emerged as a widely influential endocrine player since the expression of the CYP19 gene coding for the P450 aromatase, the enzyme responsible for the irreversible conversion of androgens into estrogens, has been detected in numerous body tissues [56].

P4 and E2 exert their effects through two main mechanisms of action. The genomic or classical mechanism involves hormone binding to an intracellular receptor, which than dimerizes and binds as a transcription factor to the P4 or E2 responsive elements (PRE or ERE) on the promoter of target genes. The non-genomic or non-classical mechanism, however, takes place in a rapid fashion, where within minutes, it triggers the activation of ion channels, second messengers, and kinases, prior to any genomic effect. Additionally, the non-classical mechanism has been associated with steroid hormone interactions with membrane-bound molecules. Membrane binding was shown by: (1) obtaining biological responses to treatment with albumin-bound hormone, and (2) showing ovarian steroid hormone-induced response by cells lacking expression of classical intracellular receptors. Furthermore, the rapid responses to sex steroids were not influenced by the inhibition of transcription or translation [57]. This further distinguished the non-classical from the classical mechanism of action, since the later involves nuclear translocation of the ligand-dependent receptor/transcription factor and genomic effects.

### 2.1. Progesterone Receptors and Mechanism of Action

The classical P4 receptor protein (PR) is a member of the nuclear/steroid hormone receptor (SHR) family of ligand-dependent transcription factors. The PR is a modular protein composed of three domains: a C-terminal ligand-binding domain (LBD), a central DNA-binding domain (DBD), and an N-terminal domain (NTD), which is considered an intrinsically disordered protein (ID) region. The PR gene (PGR) is transcribed into two isoforms: PRA (truncated NTD) and PRB (full-length). Additional isoforms have also been identified [58,59]. Biological actions of P4 depend on the expression of co-regulatory proteins and other transcription factors that interact with PR. Co-regulatory proteins interact with PR through binding to either an AF1 transcriptional activation domain in the NTD, or an AF2 domain in the LBD. Individual domain activity, and consequently, overall PR activity is also regulated by intramolecular interactions, where ligand, DNA, or co-regulatory protein binding to a given domain may impact the structural conformation and activity of other domains. In addition, ID regions in the NTD and the short hinge region, between LBD and DBD, adds further flexibility to the dynamic range of conformational changes that may take place in the receptor, regulating PR ability for interacting with other molecules. For example, the mechanism of action of PR upon binding to P4 involves induction of conformational changes to the LBD, specifically at the AF2 site, that will recruit co-regulatory proteins to activate or repress transcription. PR activity is also regulated by post-translational modifications, such as phosphorylation, ubiquitination, sumoylation, methylation, and acetylation. Modifications influence PR transport into the nucleus, dimerization and degradation, DNA binding capability, target gene-dependent induction or suppression of transcription, timing of activation of early response genes, and kinetics of DNA binding [60,61]. Evidence of membrane-associated P4-induced, non-classic, intracellular signaling soon led to the discovery of a membrane PR, first identified by Zhu et al., 2003 [62]. Currently, five members of the progestin and adiopoQ receptor (PAQR) family have been described in humans: PAQR7/mPRα, PAQR8/mPRβ, PAQR6/mPRδ, PAQR5/mPRγ, PAQR9/mPRε [63], all encoded by different genes [64]. Most recent data indicate that mPRs are molecules composed of seven transmembrane domains, an extracellular amino-terminal domain, and an intracellular carboxyl-terminal domain, suggesting they are G-protein coupled receptor-like proteins [64,65]. Membrane PRs have been identified originally in fish, and subsequently in sheep, pig, mouse, rat, and human [62,65,66,67,68]. More specifically, mPRα is mainly expressed by reproductive organs, whereas mPRβ, mPRδ, and mPRε are expressed in neural tissues, and mPRγ expressed in the urinary, digestive, and respiratory tracts [65,69]. Membrane PR gene has been reported as expressed by the endometrial and myometrial tissues, and under the regulation of E2 and P4 [70,71]. In addition, progesterone receptor membrane component 1 (PGRMC1), which is composed of an extracellular N-terminal domain, a transmembrane domain, and a cytoplasmic domain with three Src homology domains that interact with downstream signaling molecules [72], and PGRMC2, have also been implicated in P4 signaling through membrane-bound molecules.

### 2.2. Estradiol Receptors and Mechanism of Action

Similar to the PR mechanism of action, E2 activation of estrogen receptor (ER) was first defined to trigger a classical or genomic mode of action. In that context, ligand-bound ER modulates transcription either by binding directly to the estrogen response element (ERE), recruiting co-regulatory proteins, and driving transcription of target genes, or by interacting with other transcription factors (e.g., AP-1 and SP-1) [73,74,75]. There are two classical ERs: ERα, which has been cloned in the 1980s [76,77], and ERβ, identified a decade later [78]. They are transcribed from two different genes (ESR1 and ESR2) into ERα and ERβ. Within the reproductive system, ERα appears to be the predominant subtype [79] while ERβ plays a subordinate role, with exception of the ovary [80,81], the mammary gland [82], and the embryo [83]. In these systems, ERβ is necessary for mediating estrogenic action. ER protein is composed of the following domains: N-terminal region containing domains A/B, where a hormone-independent AF-1 transcriptional activation function is present; C domain corresponding to the DBD, containing two zinc fingers, and involved in receptor dimerization; D domain behaves as a flexible hinge region, and contains a nuclear localization signal (NLS); E domain refers to the LBD, where an AF-2 is located along with another dimerization site, an additional NLS, and a repression function; the F domain, at the C-terminal region, plays an additional regulatory role of receptor activity [54,56,75]. In addition to the well-established induction of genomic effects by E2, early studies reporting E2-induced acute cAMP production suggested a non-classical or non-genomic mechanism of action [84]. Currently, it is accepted that the non-classical mechanism involves direct triggering of second messenger molecules and acute regulation of intracellular signaling after binding to classical cytoplasmic or plasma membrane-recruited ERα, and ERβ to a lesser extent [85,86,87,88]. In addition, experiments performed in cell systems lacking evidence of classical ER-derived activity allowed the identification of membrane-associated E2 binding sites, which were later identified as a G-protein coupled receptor (GPR30), initially classified as an orphan receptor [89,90,91]. Later, the acute regulation of cell signaling was linked to GPR30, which was then renamed to G protein-coupled estrogen receptor (GPER) [92,93].

## 3. Sex Steroids Dictate Oviductal Function

The mammalian oviduct undergoes significant morphological [45,94,95], biochemical [96,97], and physiological [98,99,100] changes during the estrous cycle. These changes are mainly mediated by endocrine mechanisms specifically controlled by the ovarian steroids, E2 and P4. Early studies had already drawn the attention for the functional regulation of oviduct biology by E2 and P4. Since 1956, E2 has been shown to stimulate oviduct fluid secretion, and to rescue fluid secretion in ovariectomized rabbits, whereas P4 showed a suppressive effect [97,101,102]. In 1969, Brower and Anderson described the histological changes observed in the rabbit oviduct throughout the estrous cycle, suggesting an ovarian sex steroid endocrine control [1]. Authors presented evidence for the release of secretory material from the oviductal epithelium at a stage that matches the transit of early cleavage embryos through the oviduct. Moreover, McDaniel et al. [103] studied the influence of E2 and P4 on the histology and histochemistry of the bovine oviduct. After using samples from ovariectomized cows supplemented with E2, P4, and their combination, authors showed that secretory products were induced by E2 and inhibited by P4. Later, additional cellular activities were shown to be controlled by ovarian sex steroids: motility [104], ciliation and secretion [105], oocyte transport [27], and sperm transport [106]. This early evidence provided strong support for the hypothesis describing the oviduct as a dynamic organ, which plays relevant roles in the reproductive biology context under the regulation of E2 and P4. More recently, studies demonstrating the expression of ovarian sex steroid receptors in the oviduct support the regulation of oviductal function by E2 and P4. According to Ulbrich et al. [107], ESR1, ESR2, and PGR transcripts and proteins have been detected in bovine oviduct epithelial cells, and their expression varies in both the ampulla and the isthmus according to the phase of the estrus cycle. Briefly, at the mRNA level, ESR1 and ESR2 expression in the isthmus was greater at the follicular and mid to late luteal phases, respectively, though constant in the ampulla. At the protein level, ERα, ERβ, and PR were detected in epithelial and smooth muscle cells of the oviduct. Additionally, authors reported an increased expression of ERα at early luteal phase, reduced expression of ERβ at follicular phase, and increased expression of PR at the follicular phase. ERα expression was greater in the ampulla, and that of ERβ in the isthmus. Furthermore, overall PR protein was induced in the follicular phase in the isthmus and ampulla, but PRA showed a stronger signal in the ampulla, whereas PRB was more intense in the isthmus. In bovine oviduct epithelial cells, P4 induced ERβ gene expression, while E2 induced ERα and PR expression [107]. Similar results were reported by Valle et al. [108], who identified a positive correlation between circulating concentrations of E2 and P4, and ER immunostaining and a negative correlation of PR immunostaining with P4 circulating concentrations. 

### 3.1. Sex Steroids Control Oviductal Gene Expression

A summary of sex steroids-mediated changes in gene and protein expression is presented in Table 1. Numerous transcriptional profiling studies were performed aiming to connect upstream regulators, downstream pathways and biological activity, and establish molecular signatures of the functional regulation of key segments of the reproductive tract. In the 2000s, Bauersachs and coworkers conducted pioneering work to characterize the oviductal transcriptome using microarray technology. They compared global gene expression between oviducts ipsilateral and contralateral to the dominant follicle at estrus and diestrus. In the first study, 35 differentially expressed genes (DEG) between ipsilateral and contralateral oviducts were identified. From those, 27 genes were expressed at a higher level in the ipsilateral oviduct [109]. The ipsilateral-induced genes or their products control important molecular pathways, such as calcium binding, lipid transport, cell–cell interaction, nucleotide and polyamine metabolism, and cell signaling, among others. The genes upregulated in the contralateral side were related to extracellular matrix (ECM) proteins and immune functions, among others. Authors concluded that critical local regulatory mechanisms of oviduct epithelial function could be mediated by the proximity to the ovary and/or the presence of a cumulus–oocyte complex. In their second study, bovine oviductal samples from days 0 (estrus) or 12 (diestrus) were assessed by microarrays to determine the influence of the estrous cycle phase on gene expression profile [110]. Seventy-seven DEG were identified, further supporting the oviduct regulation throughout the estrous cycle, most likely due to the fluctuation of sex steroid concentrations. Genes involved in the regulation of protein secretion and modification, as well as mRNAs of secreted proteins were upregulated during estrus. During diestrus, the expression of genes involved in transcriptional regulation was greater. Both these studies represented a change of paradigm for all the molecular studies in oviductal biology, as they showed the existence of spatial-specific and temporal-specific regulation of the oviduct response to ovarian sex steroids. It was next postulated that unique histological architecture and biological roles of the oviductal segments could result from a distinct pattern of exposure to different concentrations of ovarian sex steroids associated with a distinct blood vessel supply [47,48]. In 2015, Cerny et al. [111] used microarray to compare the transcriptome of the ampulla and isthmus during the follicular and luteal phases. The oviductal regions presented different transcriptome profiles (ampulla vs. isthmus; 1569 DEG), which varied in response to the phase of the estrous cycle (follicular vs. luteal; 1763 DEG). The molecular enrichment analysis of ampulla upregulated genes showed greater representation of biological processes, such as cell cycle, cholesterol biosynthetic process, cell division, mitosis, and protein folding during the follicular phase. In the isthmus, the most representative biological processes during the follicular phase were protein folding, cell cycle, cell division, mitosis, and electron transport chain. The authors concluded that there were both spatial- and temporal-related changes in the transcriptome of the ampulla and isthmus epithelium. More recently, Gonella-Diaza et al. [112] used RNAseq technology to compare the oviductal transcriptome of groups of animals exposed to distinct periovulatory endocrine milieus associated with contrasting fertility in vivo [113]. Groups consisted of cows ovulating a large pre-ovulatory follicle (POF) and forming a larger CL (LF-LCL group; greater fertility) with a group of cows ovulating smaller POF and forming a smaller CL (SF-SCL group). This study provided direct evidence that oviductal transcriptome is differentially responsive to the sequential exposure to E2-P4 concentrations at proestrus–metestrus, respectively. The ampulla and isthmus of LF-LCL cows presented greater abundance of transcripts related to ECM regulation and homeostasis. Both biological processes have been associated with embryonic survival and development. Remodeling is important to release growth factors sequestered in the ECM. Homeostasis-related genes modulate the redox balance in the oviductal lumen, to which gametes and embryos are exposed. Authors concluded that functional characteristics of the oviduct are regulated by the periovulatory sex steroid milieu, and may potentially affect early embryonic development, and ultimately, fertility.

### 3.2. Sex Steroids Control Oviductal Protein Expression

Until the establishment of intimate maternal–embryo interaction, embryo development is influenced directly by the secretory products of oviductal and endometrial epithelial cells. Reproductive processes that occur in the oviductal lumen are potentially under the direct influence of the oviductal fluid and its components [37]. These components could have two different origins: molecules secreted by the luminal epithelia or molecules coming from plasma [35]. The oviductal fluid is colorless, and has a slightly alkaline pH (pH 7.7 to 8.2). The specific gravity of the fluid is less than 1.0, and osmolality is around 310 mOsm [120]. In addition, it contains many metabolites such as glucose, lactate, pyruvate, and amino acids. The concentration of these metabolites differs from those found in the uterine fluid or plasma. This implies that composition of this microenvironment is regulated locally. The large number of oviduct-specific proteins identified in the oviductal fluid corroborates the idea of local regulation [35]. Bishop et al. [97] used an experimental model in which the uterine–tubal junction was ligated, and the oviductal fluid was collected through the infundibulum in rabbits. It was observed that the oviduct produced 0.79 mL of fluid daily at a maximum pressure of 34 mmHg. In the context of ovarian sex steroid regulation of oviductal function, changes in concentrations of molecules derived from the biosynthetic activity of the oviductal cells are stimulated by E2 in a region-specific manner. In a study by Buhi et al. [121], it was shown that each oviductal region responded differently to E2, leading to different regional patterns of secretion. Using exogenous E2 to stimulate ovariectomized sows, authors observed an increase in the synthesis and secretion of macromolecules in the infundibulum and the ampulla, whereas the synthesis remained unchanged in the isthmus. A “regional gradient” pattern of macromolecules secretion was proposed, where the ampulla secreted greater amounts of macromolecules than the infundibulum, and the latter more than the isthmus. Similar results were also reported in sheep [122]. Binelli et al. 1999 [115] employed an experimental model in which oviductal explants were cultured in vitro in a medium supplemented with radiolabeled leucine. Using samples from animals treated to grow a “fresh” or a persistent dominant follicle, they determined that the de novo protein synthesis by oviductal cells varied according to the length of exposure to E2. In humans, oviductal fluid content is at its lowest protein concentration at ovulation [123]. A proteomic study of the oviductal fluid took a step further to assess the influence of the estrous cycle (estrus vs. luteal phase) on protein expression [119], and observed that 81 out of the 280 quantified proteins presented a differential pattern of expression at estrus, in comparison to the luteal phase. During estrus, the most abundant proteins were oviductin, isocitrate dehydrogenase, elongation factor 1-α1, heat shock 70 kDa protein 8 (HSPA8), 14-3-3 protein ε, and annexin A8; whereas, in the luteal phase, α-2-macroglobulin, ceruloplasmin, gelsolin, transthyretin, and complement factor B were more highly expressed.

Among the proteins synthesized by the oviductal cells that have been better characterized, the oviductal glycoprotein 1 (OVGP1) has been the most extensively studied [36,114]. The OVGP1 is similar to members of the mucin and the glycosyl hydrolase 18 gene family, in which proteins with enzymatic activity are included, although no enzymatic activity for OVGP1 has been reported. The OVGP1 gene is present in the genome of various animals, including all mammals [35]. OVGP1 was described as a class of oviduct-specific glycoproteins that was present in the luminal fluid at the time when fertilization occurs [114]. This protein is synthesized exclusively by non-ciliated secretory cells, and has been reported to interact with gametes and embryos, with maximum expression level near the peak of E2 concentrations in cows [36,37]. Employing immunomicroscopy techniques, it was demonstrated that OVGP1 can penetrate the zona pellucida and enter the perivitelline space. It also interacts with the acrosomal membrane of sperm cells [124]. However, no specific role for OVGP1 during fertilization and embryonic development has been identified, as OVPG1 knockout mice have normal fertility [125]. Additional functional studies have revealed the effect of E2 to induce glutathione peroxidase 4 (GPX4) protein expression and activity [118], and to regulate the production of antimicrobial peptides and suppress protease activity [126] in the oviduct of cattle and mice, respectively. The current thinking in the field is that, under the regulation of ovarian sex steroids, oviductal cells may establish an adequate microenvironment to nourish gametes and early developing embryos by providing protection from oxidative stress and potential embryotoxic effects of the oviductal mucosa innate immunity. 

### 3.3. Sex Steroids Modulate Morphological Characteristics and Activity of the Oviduct

A summary of the current evidence supporting sex steroid control of oviductal morphology and transport is presented in Table 2. During prenatal development of the reproductive tract, the oviductal lumen is defined as a fairly flat epithelial lining, without appreciable folding. Only in the postnatal life the complexity of mucosal folds starts to develop. In the adult animal, oviductal folds develop into a complex organization exhibiting primary, secondary, and tertiary folds [127,128,129]. At this point, the oviductal epithelium forms longitudinal folds that increase the epithelial surface area, allowing for an improved interaction with gametes and embryos during their transit through the organ. Interestingly, however, in spite of variations of the folding pattern according to the oviductal region [45,130] and the phase of the estrous cycle [103,131,132], studies have demonstrated that Esr1 and Pgr knockout mice present a normal oviductal morphology [133,134].

In general, the infundibulum and ampulla present a greater number and complexity of folds than the isthmus; additionally, during estrus, the number of folds, as well as the degree of folding is increased [132]. The oviductal epithelium contains both columnar ciliated cells and non-ciliated secretory cells, also known as peg cells [95,96,130]. Cellular populations vary across the oviductal regions, depending on the species and the day of the estrous cycle (and/or pregnancy). In general terms, the proportion of secretory cells increases towards the regions nearest to the ovaries (i.e., infundibulum and ampulla); an opposite trend is observed for ciliated cells, which populate in greater proportion the oviductal regions nearest to the uterus (i.e., isthmus and UTJ) [1,45,132,137]. 

The cilia are membrane specializations of the cellular apical domain, and have coordinated movements, which help to propel structures in the oviductal lumen [140]. Ciliated cells are involved in sperm capacitation, transport of sperm cells and embryo, and to some extent, secretion of molecules into the lumen [4,26,141]. The nucleus of ciliated cells is spherical and located in the middle of the cell. The cytoplasm is slightly basophilic and rich in mitochondria [95]. In rabbits, it was observed that the cytoplasm of ciliated cells has abundant mitochondria, ribosomes, and microtubules, which provide sufficient energy for ciliary movement [1]. It is believed that P4 concentrations can affect embryo development by regulating their transport into the uterus [8]. In cows, it has been shown that P4 reduces the motility of the oviduct in vivo, and the motility of cilia ex vivo via non-genomic signaling [137]. Non-genomic P4-induced suppression of ciliary beating was also observed in other species [142,143]. This is consistent with the presence of non-classical PR close to the ciliary stalk in oviduct ciliated cells [144]. Indeed, Nutu et al. [145] reported that mPRβ and mPRγ are expressed in the E2/P4-sensitive ciliated cells of the oviduct. On the other hand, E2 acts to accelerate oocyte transport through the oviduct, likely by stimulating PKC and PKA activity, and acts through ERα to increase cilia length and beating frequency [146,147,148]. Interestingly, in rabbits, the number of cilia/cell decreases during the follicular phase [138].

Non-ciliated cells have a known secretory activity, and may reach a larger size than ciliated cells. Unlike ciliated cells, secretory cells have their cellular machinery specifically organized for the synthesis and secretion into the oviductal lumen [1,149]. During the follicular phase, they develop characteristic cytoplasmic granules [45,140]. It is hypothesized that secretions from these cells provide nutrients for the oocyte and embryo [46,150], and could favor sperm capacitation [26,151]. Moreover, it was established that the period of time in which oocytes are in transit through the ampullary lumen coincides with the days when secretory cells exhibit morphological changes indicative of increased activity [136]. Secretory cells are more basophilic and electron-dense than ciliated cells, and could have a columnar or irregular morphology [95]. The apical surface of the secretory cells may be positioned at a similar or greater height than that of ciliated cells, forming cellular protrusions in the lumen of the oviduct [95]. After ovulation, shorter secretory cells are commonly observed; however, cells tend to increase in size and number of cytoplasmic protrusions throughout the reproductive cycle [95], suggesting a relationship between secretory cell phenotype and endocrine fluctuations of the estrous cycle [96]. Protrusions may contain rough endoplasmic reticulum (RER) portions, mitochondria, and ribosomes. The nucleus of the secretory cell is located near the base of the cell, has an irregular shape, and is generally surrounded by RER. In electron microscopy micrographs, the secretory cell is electron-dense due to the abundance of RER and free ribosomes in the cytoplasm [1]. In 1995, Murray [135] described pregnancy-associated morphological changes in the oviduct, specifically in protein-synthesizing organelles in secretory cells. In addition, secretory products contained in cytoplasmic granules are released by the process of exocytosis until Days 3 and 4 after ovulation, and cell death appears to occur at Day 16 by shedding of epithelial cells into the oviduct lumen. A recent study conducted by Ghosh et al. [152], where an experimental model based on mouse in vivo genetic cell lineage tracing was used, showed that secretory cells not only self-renew, but also give rise to ciliated cells. Also, Ito et al. [153] concluded that the proportion of ciliated and secretory epithelial cells changes during the estrous cycle, and that at least in cattle, only the secretory cells proliferate. In summary, remodeling of the bovine oviductal epithelium represents a necessary step in the process of preparing an optimal environment for gamete transport, fertilization, and embryonic development. Remodeling is mainly regulated by proliferation, differentiation and apoptosis of secretory cells, and these processes seem to be regulated by estrous cycle-related endocrine changes. It is expected that adequate remodeling is required to ensure appropriate maternal receptivity [154,155,156]. In this context, by using an animal model that modulated the periovulatory endocrine milieu by controlling the size of the preovulatory follicle [157], our research group was able to show that cows ovulating a larger follicle, which generated a larger CL, and presented greater E2 and P4 concentrations (proestrus and early diestrus, respectively), undergo more dramatic oviductal remodeling [139]. More specifically, oviducts had more primary mucosal folds, greater mucosal folding grade, luminal perimeter, more secretory oviductal cells, and a greater oviductal cell proliferative activity [139]. Consistently, the periovulatory endocrine profile associated with greater remodeling has also been associated with greater fertility [113].

## 4. Conclusions

**Figure 2:**
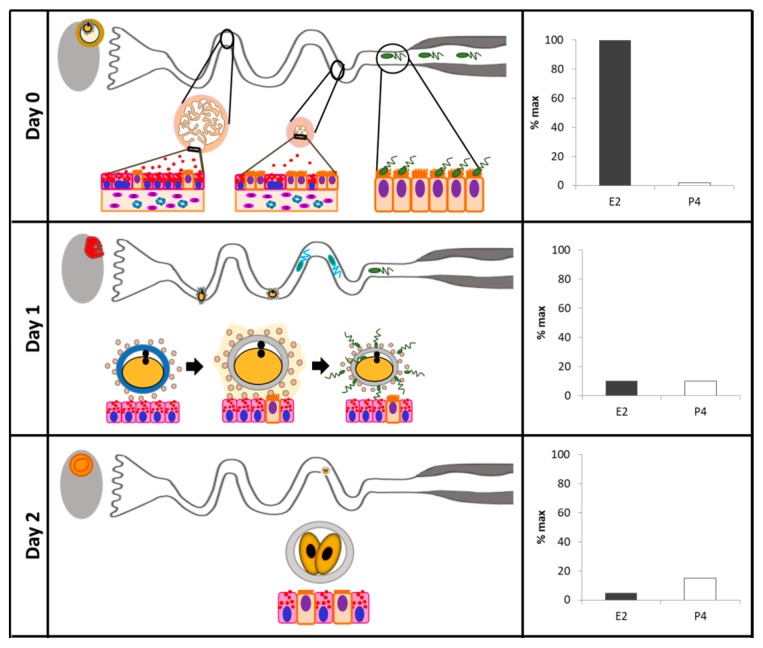

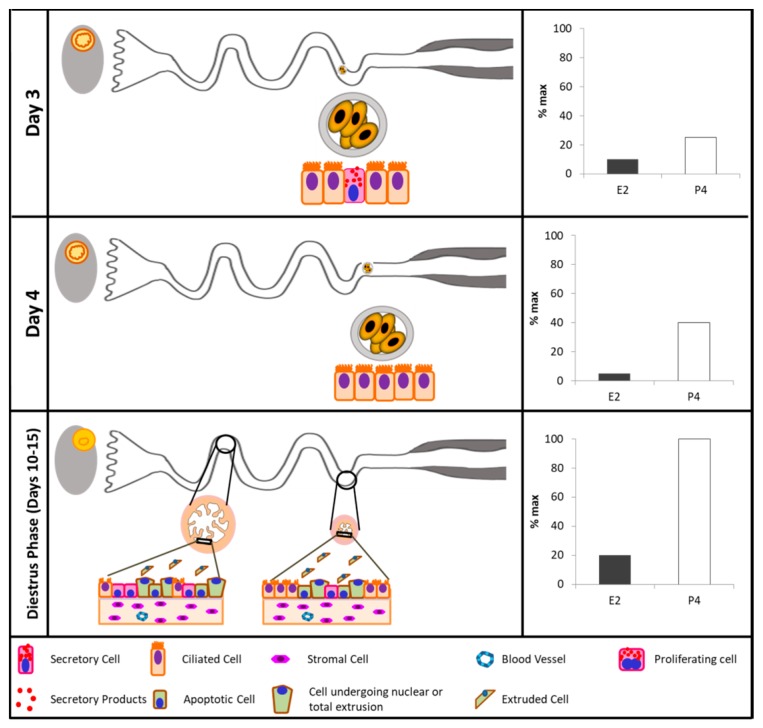
Reproductive events that occur in the oviduct in association to temporal changes in sex steroid concentrations. In each panel, the expected localization of gametes or the embryo is represented in the context of the reproductive processes that are ongoing, in association with oviductal cells, for each day of the cycle. On the left of each panel, the bar graph shows relative concentrations of E2 and P4, represented as a percentage of the maximal concentration of each hormone achieved during the estrous cycle. Day 0 (standing estrus): during the pre-ovulatory phase, the pre-ovulatory follicle produces maximal concentrations of E2. In the ampulla, the tunica mucosa became more folded, the luminal epithelium is taller and, specifically, the secretory cells secrete macromolecules via secretory granules to the oviductal lumen. In the isthmus, the ciliated cells gain more cilia, and ciliary beating increases. In both regions, proliferation of secretory cells and vascularization is observed. After mating, spermatozoa migrate and accumulate in the caudal portion of the isthmus, bound to ciliated cells. Day 1 (ovulation): under decreasing E2 concentrations, the cumulus–oocyte complex (COC) is transported from the infundibulum to the site of fertilization in the ampulla within 30–45 min. At the site of fertilization, cumulus cells establish a strong connection with the oviductal epithelial cells, which pauses COC movement. Meanwhile, the sperm cells are released from the isthmus, resume their cranial migration, and begin hyperactivation and early acrosome reaction. Fertilization ensues. Day 2: as soon as a sperm cell penetrates the zona pellucida, the zygote detaches and continue its caudal migration. Embryonic cells undergo mitosis and the embryo grows. Decreasing concentrations of E2 and increasing concentrations of P4 decreases the speed of embryo transport. This allows exposure of the embryo to ampullary secretions that may affect its composition and development. Day 3: as oviductal secretions affect the development of the embryo, it in turn modulates the oviductal transcriptome and secretome in a complex two-way communication. The oviductal fluid is the sole extraembryonic source of nutrients and growth factors that the embryo needs to continue developing. As the embryo transitions to the isthmus, it finds an increasing proportion of ciliated cells. Day 4: final transport of the embryo to the uterus occurs around 3.5 days after fertilization, under increasing concentrations of P4. Diestrus phase (Days 10–15): during the luteal phase, luteal P4 inhibits oviductal secretory and transport activities. Folding of the ampullary tunica mucosa, height of luminal epithelium, and quantity of secretory cells is reduced. Secretory cells extrude their nuclei. In the isthmus, there is less quantity and activity of cilia. In both regions, there is clear evidence of apoptosis and partial or total cellular extrusion, and there is less vascularization. These processes are associated with epithelial desquamation and renewal, as the oviduct prepares for the next estrous cycle.

On a temporal scale, within a window of time when major sexual endocrine events take place (i.e., the periovulatory phase), the oviduct undertakes its most important role: to establish physicochemical interactions with gametes and early embryos, and influence their final maturation and initial developmental, respectively. Solid evidence exists supporting the regulation of oviductal function by ovarian steroids towards the transport of gametes and the embryo, by influencing ciliary beating, smooth muscle contraction, and secretory activity. While under the E2 stimulus, the oviducts increase their secretory capacity, transport velocity, and cellular proliferation; under P4 stimulus, the oviducts reduce their transport velocity and secretion, and start a process of self-renewing the epithelium for the subsequent estrous cycle (Figure 2). Expanding the investigation towards mechanistic processes integrating ovarian steroids, intracellular signaling pathways, and the regulation of the oviduct luminal environment, is a necessary step towards the appropriate comprehension of oviduct biology. It is important to understand the fine-tuning control that E2 and P4 exert on oviductal physiology, in order to improve the in vitro culture conditions and to obtain better quality embryos.

## Figures and Tables

**Table 1:** Summary of evidence of sex steroid control of oviductal gene and protein expression.

Species	Studied Effect	Sample Type	Phase of the Cycle	Method Used	Sex Steroids-Mediated Effect	Source
Cattle	Characterization of Glycoproteins	Tissue sections of ampulla and isthmus	Days 0, 5, 10, 15, and 18	Electrophoresis and fluorography, Western blot, light microscopy, and colloidal gold immunolabeling	The cattle oviductal epithelium synthesizes and secretes a class of oviduct-specific glycoprotein that is present in luminal fluid at the time when fertilization occurs.	Boice et al., 1990 [114]
Cattle	Oviductal secretory proteins in cows with persistent dominant follicle	Infundibulum, ampulla and isthmus explants	Cows with fresh or persistent dominant follicle (estrus)	In vitro culture of explants, two-dimensional electrophoresis, densitometry	Rate of incorporation of [3H]leucine into macromolecules was greater in the infundibulum, ampulla, and isthmus of cows with a fresh dominant follicle.	Binelli et al., 1999 [115]
Cattle	Expression of β2-adrenergic receptors	Oviductal epithelial cells collected after flushing the oviductal lumen.	Secretory, early to midluteal, proliferative, and preovulatory phases	cDNA cloning, in vitro autoradiography	P4 upregulated the β2-adrenergic receptor.	Einspanier et al., 1999 [116]
Cattle	Expression of E2 and P4 receptors in vivo and in vitro	Ampulla and isthmus epithelial cells	Early-luteal, mid-luteal, late-luteal, and follicular stages	PCR, Western blot, and immunohistochemistry	PR and ER mRNA transcripts were elevated in vivo during the follicular phase. The highest PR and ER protein expression was detected during the early-luteal phase. In vitro, E2-supplementation resulted in an upregulation of PR and ER.	Ulbrich et al., 2003 [107]
Cattle	Tumor necrosis factor expression during the estrous cycle	Whole oviductal tissue sections	Follicular, postovulatory and luteal phases	In vitro microdialysis in organ culture chamber and qPCR	Infusion of TNFa stimulated oviductal secretion of PG, ET-1, and Ang II during the follicular and postovulatory stages, but not during the luteal stage. High expression of both TNFaR types and ligands was detected during the follicular and postovulatory stages, whereas low expression was detected during the luteal stage.	Wijayagunawardane et al., 2003 [117]
Cattle	Comparison of ipsilateral vs. contralateral to corpus luteum (CL) oviducts	Oviductal epithelial cells	Day 3.5	Subtraction cDNA libraries and cDNA array hybridization.	35 cDNAs differentially expressed. The regulated genes or their products include a variety of functional classes such as cell-surface proteins, cell–cell interaction proteins, members of signal transduction pathways, immune-related proteins, and enzymes.	Bauersachs et al., 2003 [109]
Cattle	Comparison of oviductal samples of estrous vs. diestrus phases	Oviductal epithelial cells	Day 1 vs. Day 12	cDNA libraries and cDNA array hybridization	77 differently expressed cDNAs. Thirty-seven were expressed at a higher level at estrus. During estrus genes involved in the regulation of protein secretion and protein modification were upregulated, whereas during diestrus, particularly, transcripts of genes involved in transcription regulation were upregulated.	Bauersachs et al., 2004 [110]
Cattle	Expression of phospholipid hydroperoxide glutathione peroxidase (GPx-4)	Tissue fragments of isthmus, ishtmic-ampullary junction, and ampulla	Day 16 after E2 intrauterine infusion	In situ hybridization, qPCR, and activity assay for GPx-4.	GPx-4 expression was 2-fold higher in the oviducts of cows treated with uterine infusions of E2.	Lapointe et al., 2005 [118]
			Postovulatory, mid-luteal, late luteal, and follicular stages		There is a cell-specific distribution for GPx mRNAs in the different oviduct segments. GPx-4 expression was highest during the follicular, postovulatory, and late luteal stages.	
Cattle	Expression of E2 and P4 receptors in natural and superovulated estrous cycles	Tissue sections of infundibulum, ampulla, ampullary/isthmic transition, and isthmus of both oviducts	For natural estrous cycle, samples were collected at 17 h, 4, and 11 days after estrus. In the superovulatory treatment, samples were collected 17 h and 4 days after estrus	Immunohistochemistry	There is a positive correlation between circulating concentrations of E2 and P4 and ER staining. There is a negative correlation of PR staining with P4 circulating concentrations. There was no effect of superovulation.	Ribeiro Valle et al., 2007 [108]
Cattle	Expression of P4 receptors (nuclear and membrane components) of cyclic and pregnant cows	Sections of the whole oviduct	1.5, 4, and 5 days post-ovulation	RT-PCR, qPCR, Western blot, and immunohistochemistry	No obvious differences in localization patterns of PR, PGRMC1 and PGRMC2 were observed between ipsi- and contralateral oviducts or according to the stage post-ovulation.	Saint-Dizier et al., 2012 [8]
Cattle	Gene expression in oviductal samples of follicular vs. luteal phases	Epithelial cells from ampulla or isthmus	36 h after PGF vs. Days 11 and 12	Microarray	972 and 597 transcripts in the ampulla and 946 and 817 transcripts in the isthmus were up- and downregulated in the Follicular phase compared to Luteal phase. Upregulated genes were involved in cholesterol biosynthesis and cell cycle pathways, while downregulated genes were found in numerous inflammatory response pathways.	Cerny et al., 2015 [111]
Cattle	Large vs. small pre-ovulatory follicle	Ampulla and isthmus ipsilateral to the ovary containing the dominant follicle	Day 4	RNA sequencing (RNAseq), ERa and PGR immunohistochemistry	There was a greater abundance of PGR and ERa in the oviducts of cows having a Large preovulatory follicle. 325 and 274 transcripts were upregulated in the large follicle group, for ampulla and isthmus, respectively.	Gonella-Diaza et al., 2015 [112]
Sheep	Oviductal proteome	Oviductal fluid	Day 0 vs. Day 10	In-gel digestion coupled with mass spectrometric analysis (GeLC-MS/MS)	The proteins more abundant at estrus included several families such as the heat shock proteins, mucins, complement cascade proteins, and several redox enzymes. The proteins more abundant during the luteal phase were associated with the immune system and tissue remodeling.	Soleilhavoup et al., 2016 [119]

**Table 2:** Summary of evidence of sex steroid control of oviductal morphology and transport.

Species	Studied Effect	Sample Type	Phase of the Cycle	Method Used	Sex Steroids-Mediated Effect	Source
Cattle	P4 effect of oviductal cytology	Tissue sections of infundibulum, ampulla, and isthmus	Estrus and early postestrus, early and middle luteal phase, late-luteal phase, and proestrus-follicular phase.	Frozen sections stained with calcium-cobalt method, methyl greenpyronin Y, Best’s carmine staining for glycogen, |, alcian blue, and toluidine blue	Cell heights and cytoplasmic content of ciliated and secretory cells in ampulla and isthmus increased in the presence of E2 and decreased in the presence of P4. In luteal phase or P4-treated animals, secretory cells had extruded nuclei and epithelial desquamation.	McDaniel et al., 1968 [103]
			Ovariectomized animals treated with E2, P4, or E2 + P4			
Cattle	Oviductal motility	Recordings of electrical activity	Diestrous, proestrus, estrous, and metestrus	Electrical activity was directly recorded on a polygraph	Electrical activity start to increase at proestrus and was more intense (amplitude and frequency) at estrus and metestrus.	Ruckebusch & Bayard, 1975 [104]
Cattle	Effect of supplementation with P4 or E2 on oviductal transport	Oviductal flushing	60 h post-estrous	The oviduct was cut into 8 equal segments; each one was flushed with 1 mL of saline. Flushings were evaluated to determine the presence of an oocyte or zygote	The transport rate was increased by P4 but not affected by estradiol benzoate.	Crisman et al., 1980 [27]
Sheep	Ampullary epithelium morphology during early pregnancy	Tissue section of the ampulla	Days 0, 1.5, 2, 3, 4, 6, and 16 of pregnancy	Toluidine blue staining and electron microscopy	The ampullary secretory cells undergo morphological alterations in protein-synthesizing organelles and apical specializations that vary with the stage of pregnancy.The secretory products contained in cytoplasmatic granules are released by the process of exocytosis until Days 3 and 4. Cell death appears to occur at Day 16 by shedding of epithelial cells into the oviductal lumen.	Murray, 1995 [135]
Rat	Cyclic changes in the ampulla	Tissue sections of the ampulla	Estrus, metestrus, diestrus-1, diestrus-2, and proestrus	Electron microscopy	The days in which oocytes can be found inside the ampullary lumen are the same days when the ampullary secretory cells exhibited morphological changes indicative of increased activity.	Shirley & Reeder, 1996 [136]
Mice	Secretory cells in adult or prepubertal E2-treated mice	Oviductal tissue fragments	Proestrus, oestrus, metestrus, diestrus	Transmission electron microscopy	The proportion of secretory cells and production of secretory products increased around and after ovulation in cycling animals. E2 administration accelerates the differentiation and maturation of the secretory cells.	Lauschová, 2003 [120]
Cattle	Ciliary transport, gamete interaction, and effects of the early embryo	Ex vivo analyses with digital video microscopic system	Days 2.5, 3.5, and 4.5 of pregnancy.	Video microscopy	There are secretory and ciliated cells in the ampullary epithelium. Entering the isthmus, secretory and ciliated cells are observed. In the end portion of the isthmus, only ciliated cells are present in the oviductal epithelium.	Kolle et al., 2009 [137]
Rabbit	Oviductal morphology	Tissue sections of ampulla and isthmus	Estrus and luteal stages	Mallory’s triple stain, PAS, Alcian blue, PAS/Ab reaction, Aldehyde fuchsin/Alcian blue (AF/Ab), and electron microscopy	The numbers of secretory cells and the amount of secretions increases in the estrus stage.The amount of secretions and the number of cilia were demonstrated to decrease evidently in the luteal stage.	Özen et al., 2010 [138]
Buffalo	Oviductal morphology	Tissue sections of infundibulum, ampulla, and isthmus.	Follicular vs. luteal phase	Haematoxylin and eosin, verhoffe, toluidine blue, and PAS staining methods.	The ampulla epithelium is highly folded, taller, and presents more cells with secretory activity at the follicular phase. There is no difference in the microscopic structure of the isthmus at follicular and luteal phases. The thickness of tunica muscularis at luteal phase is significantly greater than the follicular phase in all regions.	Ayen et al., 2012 [96]
Cattle	Oviductal cell and tissue morphology	Tissue sections of infundibulum and ampulla	Luteal and follicular phases	Harris haematoxylin and eosin, van Gieson resorcin fuchsin, Goldener’s trichome stain, alcian blue stain, PAS stain, Sudan black, Gomori lead nitrate, toluidine blue staining	The secretory cells were predominant in the luteal phase with numerous apical cytoplasmic protrusions. In the ampulla the number of mucosal folds and length of the primary folds were greater at the follicular phase	Mokhtar, 2015 [131]
Cattle	Oviductal cell and tissue morphology	Ipsilateral and contralateral isthmus and ampulla	Day 4	Hematoxylin and eosine staining, PAS staining, and Ki67 immunedetection.	Animals that ovulated a large preovulatory follicle presented more primary mucosal folds, a greater mucosal-folding grade and luminal perimeter, and more secretory and proliferating cells.	Gonella-Diaza et al., 2017 [139]
